# Safety and Efficacy of Flexible Ureterorenoscopy Surgery: Results of Our Large Patient Series

**DOI:** 10.7759/cureus.23307

**Published:** 2022-03-18

**Authors:** Kadir Karkin, Ergün Alma, Ediz Vuruşkan, Güçlü Gürlen, Umut Ünal, Hakan Erçil, Zafer Gökhan Gürbüz

**Affiliations:** 1 Urology, Adana City Training and Research Hospital, Adana, TUR

**Keywords:** pelvis stone, flexible ureteroscopy, retrograde intrarenal surgery, kidney stone, kidney

## Abstract

Introduction: The aim of our study is to evaluate the success rates of our retrograde intrarenal surgery operations and the complications we encountered and to determine in which kidney segment the operations were more successful with flexible ureterorenoscopy.

Methods: The records of retrograde intrarenal surgery operations performed between March 2013 and January 2021 in Health Sciences University, Adana City Training and Research Hospital, urology clinic were analyzed retrospectively. Patients’ age, body mass index, operation side, stone size, stone density, duration of operation, first-day and first-month operation success status, presence of preoperative and postoperative ureteral stent, preoperative and postoperative first-day and first-month creatinine levels, and preoperative and postoperative first-day and first-month hematocrit levels were recorded.

Results: Our study consisted of a total of 1128 patients, 618 males (54.7%) and 510 (45.2%) females, with an average age of 42.3±14.4. Kidney stones were most commonly found in the renal pelvis (54.2%). The postoperative first-day success rate was highest in the pelvis stone group (P=0.009). The first month’s success rates were highest in those with pelvic stones (93.1%), and the lowest in patients with multiple stones (85.7%). Patients’ operation time, postoperative hematocrit and creatinine levels, and complications did not differ statistically between the groups (P>0.05).

Conclusion: Retrograde intrarenal surgery is an acceptable minimally invasive and effective surgery with low complication rates. There is a high success rate, especially in pelvis stones.

## Introduction

Different methods are used in the surgical treatment of kidney stones. Today, shockwave lithotripsy (SWL), percutaneous nephrolithotomy (PNL), and retrograde intrarenal surgery (RIRS) are commonly used treatment options. Open kidney stone operations that have played an important role during the historical process, now have been abandoned. PNL and RIRS are safe and effective treatment options with high success rates, low secondary treatment needs, and acceptable complication rates [1,2]. Today, in parallel with the advancements in imaging methods and laser technology, the rate of using RIRS as an effective treatment method in minimal invasive treatment of kidney and upper ureter stones is increasing. RIRS is effective in kidney stones with a size of 10-20 mm and is recommended by European Association Urology (EAU) guidelines [3]. When considered in general, since RIRS is minimally invasive, it is a treatment method with advantages such as short hospitalization, minimal blood loss, and early return to daily activities [1]. Although RIRS is an effective surgical method in all segments of the kidneys, deflexion of the flexible ureterorenoscopy may be limited in some localizations due to the effect of laser fiber and may result in a decrease in success rates because of the inability to provide sufficient access, especially to the lower pole stones [4]. In this study, we aimed to evaluate success rates and complications we encountered with RIRS that we performed in our clinic, in line with the literature, and to determine in which kidney segment the operations were more successful with flexible ureterorenoscopy.

## Materials and methods

After receiving local ethics committee approval, we reviewed the records of RIRS operations performed in the urology clinic of Health Sciences University, Adana City Training and Research Hospital between March 2013 and January 2021. All operations were performed under general anesthesia in the lithotomy position. For the operations, 9.5/11.5 Fr urethral access stealth ve 7.5 Fr flexible ureterorenoscopy (Karl Storz, Flex x2, Tuttlingen, Germany) was used. Pediatric age group (<18 years), patients with a history of previous renal or urethral surgery, ureteropelvic or ureterovesical stenosis, elevated serum creatinine (>2 mg/dL), those with urinary system anatomic anomalies (horseshoe kidney, pelvic kidney), and patients with non-opaque kidney stones were excluded from the study. When evaluated according to the exclusion criteria, a total of 1128 patients were included in the study. Patients’ age, body mass index, operation side, stone density, operative time, first-day and first-month operation success status, presence of preoperative and postoperative urethral stent, preoperative, postoperative first-day and first-month creatinine levels, and postoperative first-day and first-month hematocrit levels were recorded. A successful operation was defined as the absence of residual stone or presence of <3 mm clinically insignificant residual fragments, while an unsuccessful operation was defined as the presence of ≥2 mm residual stones with postoperative imaging methods or a need for additional treatment (ureterorenoscopy, SWL). The patients were divided into five groups according to the stone localization as upper, middle, lower, pelvis, and multiple and evaluated. All operations were performed while urine was sterile. In the preoperative period, unenhanced computed tomography (CT) and kidney ureter bladder (KUB) graphy were used to evaluate the stone size. In patients with multiple stones, the stone area was calculated on KUB graphy for the mean stone size. Patients were evaluated with KUB graphy on the postoperative first day. Whereas, the patients were evaluated with KUB graphy and urinary system ultrasonography after 1 month of surgery. Unenhanced CT scans were performed in the patients in whom no adequate evaluation could be done.

Statistical analysis

Statistical analysis was performed using Statistical Package for Social Sciences (SPSS) 20 software (SPSS Inc., Chicago, IL). The Shapiro Wilk test was used to assess the conformity of the data to normal distribution and all normally distributed data were presented as mean ± standard deviation (SD). The student’s t-test was used for parametric variables, and the Mann Whitney U-test was used for nonparametric variables. A value of P<0.05 was considered statistically significant.

## Results

This study included a total of 1128 patients with 618 (54.7%) being male and 510 (45.2%) female, and the mean age was found as 42.3±14.4 years. No statistically significant difference was found between the groups in terms of age and gender, respectively (P=0.988, P=0.119). The kidney segment with the most common stone in our patients was determined as the renal pelvis (54.2%). Distribution of the pelvis stones for the right and left sides was found as 49.0% and 51.0%, respectively (P=0.218). The rate of inserting urethral DJ stent before the operation was found as 25% and stents were most commonly inserted in the patients with pelvis stones (P=0.075). Among the groups, the highest stone area was found in the patients with multiple stones, and the mean stone area was 114±53.4 mm^2^ (P=0.022). Stone density was the highest in the patients with multiple stones and the mean stone density was 958.1±247 HU (P=0.357). No statistically significant difference was found between the groups in terms of the preoperative hematocrit and creatinine levels (P>0.05, Table 1).

**Table 1:** Preoperative demographic data M: male; F: female; Hct: hematocrit

			Stone Localization					
			Lower	Multiple	Middle	Pelvis	Upper	P-value
Number			186	42	204	612	84	
Gender	M	n(%)	78(41.9)	12(28.6)	96(47.1)	378(61.8)	54(64.3)	0.119
	F	n(%)	108(58.1)	30(71.4)	108(52.9)	234(38.2)	30(35.7)	
Side	Left	n(%)	66(35.5)	24(57.1)	90(44.1)	312(51.0)	60(71.4)	0.218
	Right	n(%)	120(64.5)	18(42.9)	114(55.9)	300(49.0)	24(28.6)	
Radiopacity	Yes	n(%)	168(90.3)	42(100)	198(97.1)	564(87.3)	72(85.7)	0.439
	No	n(%)	18(9.7)	0(0.0)	6(2.9)	48(12.7)	12(14.3)	
Preoperative Stent	Yes	n(%)	84(45.2)	12(28.6)	36(17.6)	132(21.6)	18(21.4)	0.075
	No	n(%)	102(54.8)	30(71.4)	168(82.4)	480(78.4)	66(78.6)	
Age			42.3+14.4	43.7+10.8	41.9+15.9	42.2+14.5	43.0+13.2	0.998
Stone area			114+53.4	164.1+71.4	126.8+47.2	111.9+42.1	137+60.1	0.022
Density			894.7+287.7	958.1+247	860.9+263.2	862.4+266.5	735.6+277.8	0.357
Creatinine (preoperative)			0.96+0.31	1.0+0	0.97+0.17	1.0+0.17	1.0+0	0.706
Hct (preoperative)			39.6+4.6	39.2+4.6	39.2+4.2	40.4+4	40.7+4.8	0.549

The highest postoperative first-day success rate was found in the pelvis stone group (P=0.009). The highest success rate was found in the patients with pelvic stones by 88.2%, while the lowest success rate was in the multiple stones group by 57.1%. The highest first-most success rate was in the pelvis stones group (93.1%), and the lowest success rate was again in the multiple stones group (85.7%). The rate of postoperative stent insertion was 76.0% (P=0.235). The highest rate of the postoperative clinically insignificant residual stone fragment (CIRF) was found in the patients with lower pole stones (P<0.001). No statistically significant difference was found between the groups in terms of operative time, postoperative hematocrit and creatinine levels, and complications (P>0.05, Table 2).

**Table 2:** Postoperative demographic data *CIRF: clinically insignificant residual fragment; *Hct: hematocrit

			Stone localization							
			Lower	Multiple	Middle	Pelvis		Upper		P-value
First day success	Yes	n(%)	114(61.3)	24(57.1)	162(79.4)		540(88.2)	66(78.6)	0.009	
	No	n(%)	72(38.7)	18(42.9)	42(20.6)		72(11.8)	18(21.4)		
First month success	Yes	n(%)	162(87.1)	36(85.7)	198(97.1)		570(93.1)	72(85.7)	0.476	
	No	n(%)	24(12.9)	6(14.3)	6(2.9)		42(6.9)	12(14.3)		
Stent	Yes	n(%)	144(77.4)	42(100)	174(85.3)		432(70.6)	66(78.6)	0.235	
	No	n(%)	42(22.6)	0(0.0)	30(14.7)		180(29.4)	18(21.4)		
CIRF*	Yes	n(%)	102(54.8)	72(57.1)	42(20.6)		96(15.7)	18(21.4)	<0.001	
	No	n(%)	42(45.2)	9(42.9)	81(79.4)		258(84.3)	33(78.6)		
Fever	Yes	n(%)	6(3.2)	0(0.0)	18(8.8)		18(2.9)	0(0.0)	0.482	
	No	n(%)	180(96.8)	42(100)	186(91.2)		594(97.1)	84(100.0)		
Pain	Yes	n(%)	24(12.9)	12(28.6)	30(14.7)		120(19.6)	12(14.3)	0.801	
	No	n(%)	162(87.1)	30(71.4)	174(85.3)		492(80.4)	72(85.7)		
Mucosal injury	Yes	n(%)	12(6.5)	0(0.0)	6(2.9)		48(7.8)	0(0.0)	0.63	
	No	n(%)	174(93.5)	42(100)	198(97.1)		564(92.2)	84(100.0)		
Hematuria	Yes	n(%)	12(6.5)	0(0.0)	18(8.8)		66(10.8)	12(14.3)	0.801	
	No	n(%)	174(93.5)	42(100)	186(91.2)		546(89.2)	72(85.7)		
Operation time			58.4+13.6	68.2+19	56+12.3		57.7+13.3	64.7+10.9	0.082	
Creatinine (postoperative)*			1.03+0.17	1.0+0	1.0+0		1.03+0.17	1.01+0.05	0.793	
Creatinine (1-month)*			0.96+0.17	1.0+0	0.97+0.17		1.0+0	0.99+0.02	0.455	
Hct (postoperative)*			37.97+5.08	38.14+5	37.5+4.4		38.7+4.1	39.5+4.8	0.523	

## Discussion

RIRS has become a treatment method with increasing usage rate and popularity, especially in the last decade. Minimal invasiveness, performing with the use of natural orifice, low morbidity, and satisfying stone-free results increase the popularity of this method worldwide [5,6]. Of course, the effects of advancements in the technique and technology are of paramount importance in this increase. Although there are studies demonstrating that the treatment of kidney stones >2 cm with RIRS can also be safely performed, the main target patient group is those with stones of 10-20 mm who are resistant to SWL [3].

Stone localization in the kidney has an important effect on postoperative success rates. Although much thinner fibers are used with the developments in laser fiber technology, it is obvious that the rates of access to the stone and success rates may be affected due to the inability of the instrument to make adequate deflexion, especially in the interventions performed for the lower pole stones. Breda et al. reported an overall stone-free rate of 79% after the first session and 100% after the second session in the patient group with ≤2 cm stones, these rates were reported as 52% and 85.1% in the patients with >2 cm stones [7]. Resorlu et al. reported an overall success rate of 88% with RIRS and the need for additional procedures as 8.7% [8]. According to our study, the postoperative first-day success rate was 80.9% and postoperative first-month success rate was 91.5% in overall patients, and RIRS was the most successful in renal pelvis stones. That ease of access to stones and minimal effect of laser fiber on the deflexion of flexible ureterorenoscopy might play a role in this success. According to our results, our overall success rates were sufficient for all kidney segments, although the success rate was lower in the patients with lower pole and multiple stones. We think that it would be more appropriate to move the stone with a basket to another calyx in a more suitable localization and to perform laser fragmentation here. In this way, stone fragmentation is done more easily and the service life of the flexible ureterorenoscopy prolongs due to less forcing to deflexion. It is obvious that this will reduce operative costs. That low success rates in the patients with multiple stones might be caused by decreased visualization quality due to prolonged operative time and high stone areas. However, we believe that success rates obtained with these minimally invasive methods are adequately satisfying.

Although it is known that insertion of the standard ureteral stent before RIRS decreases ureteral resistance, facilitating ureteral access sheath (UAS) insertion and reducing possible ureteral injury, a debate is ongoing on whether preoperative stenting is necessary as a routine application [9,10]. In a select cohort of patients with preoperative CT urogram, Viers et al. observed a 17% incidence per patient of primary upper tract access failure necessitating presenting and a 15% incidence of presenting-related complications [11]. Rubenstein et al. reported that preoperative stenting provides passive urethral dilatation before RIRS, facilitating the passage of the ureteroscope [12]. However, routine use of preoperative stenting is not recommended by EAU [3]. Postoperative stenting decreases hydronephrosis, increases the rate of spontaneous stone fragmentation passing, and reduces ureter stricture [4]. However, it should be kept in mind that ureter stents are associated with some morbidities such as irritative symptoms, bacteriuria, and sexual dysfunction [13]. In our study, preoperative stenting was not routinely performed and the rate of preoperative stenting was 25%. The most common preoperative stenting was performed in our patients with pelvis stones (21.6%). According to our results, the first-month success rate was 93.75% in the patients with pelvis stones, and UAS insertion was performed without problem in these patients. Within this context, although preoperative stenting is seen as helpful in operative success, it should be known that a secondary operation is needed in the patients, which will cause an additional cost. While with increasing clinical experience, we do not recommend routine preoperative stenting, we routinely perform postoperative urethral stenting in all of our patients and we think that it would be appropriate to remove the stent in an office setting with local anesthesia 15 days later.

RIRS is an appropriate treatment option as a minimally invasive method and with low complication rates in patients with kidney and upper ureter stones. Breda et al. emphasized that RIRS has minimal morbidity with 3.9% intraoperative, 1.9% major, and 13.6% overall complication rates [7]. In a global study by Perez et al., the overall complication rate was reported as 3.5% and stated that most complications were grade 1 and 2 (Clavien-Dindo). Blood transfusion rate was 0.2%, while mortality occurred only in five patients due to several causes such as sepsis, pulmonary embolism, and multiorgan failure [14]. In our study, all complications were low-grade and the most commonly observed complication was postoperative pain. None of our patients developed blood loss requiring transfusion or macroscopic hematuria. A commonly recognized opinion in the literature about RIRS is that increasing surgical experience positively affects results and complications [15]. This study has some limitations. The most important limitation is the retrospective design of the study. In addition, it reflects the results of a small number of patients and shares the experience of a single center.

## Conclusions

RIRS has high efficacy and low morbidity in the treatment of kidney stones. Stone-free rate is higher in patients with pelvis location and a single stone. It has an acceptable stone-free and complication rate even in patients with stones in the lower pole and multiple stones. However, repetitive surgeries may be needed more in patients with lower pole locations and multiple stones.
